# Supplementary material for: Zbtb40 Deficiency Leads to Morphological and Phenotypic Abnormalities of Spermatocytes and Spermatozoa and Causes Male Infertility
Source: Cells. 2023 Apr 26;12(9):1264. doi: 10.3390/cells12091264 (PMC10177581; doi:10.3390/cells12091264)
Supplement: Supplementary file 1 [file cells-12-01264-s001.zip › cells-2268285-supplementary.pdf]

Supplemental Data

ZBTB40 deficiency leads to morphological & phenotypic abnormalities

of spermatocytes and spermazoa and causes male infertility

Supplemental Figures S1-S3

Figure S1. The diagram showed the sequence of *Zbtb40* knockout mice.

Figure S2. ZBTB40 expression in the *Zbtb40*$^{+/-}$ mice.

Figure S3. The influence of *Zbtb40* knockout on the number of the leptotene, zygotene, pachytene, and diplotene spermatocytes.

Supplemental Tables S1-S4

Table S1. Primers for PCR of ZBTB40 knockout mice

Table S2. Primary antibodies used in this study

Table S3. Secondary antibodies utilized in this study

Table S4. Sequences of telomeric oligonucleotides

Supplemental Figures 1-3

Figure S1

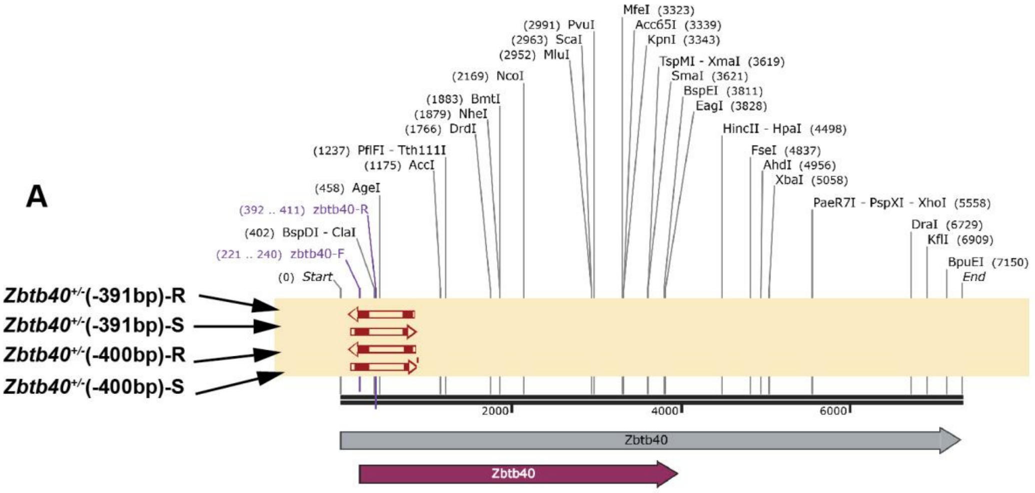

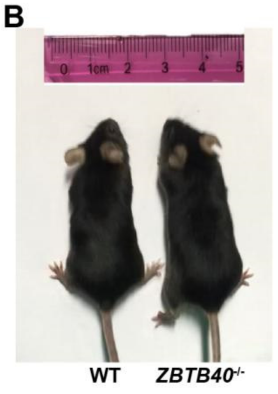

C

Parental genetype	♂ Zbtb40$^{-/-}$ (-391bp)	♀ Zbtb40$^{-/-}$ (-391bp)	♂ WT	♀ Zbtb40$^{+/-}$ (-400bp)	♂ Zbtb40$^{+/-}$ (-400bp)	♀ WT	♂ WT	♀ WT
Average cage time(day)	2.75±1.500		51.5±7.778		> 60		3.29±0.951	
Fetuses(n)	205		19		NA		298	
Utter size± stdev	7.00±2.236		6.33±0.577		NA		9.30±1.421	

Figure S1. The diagram showed the generation of *Zbtb40* knockout mice.

Figure S2

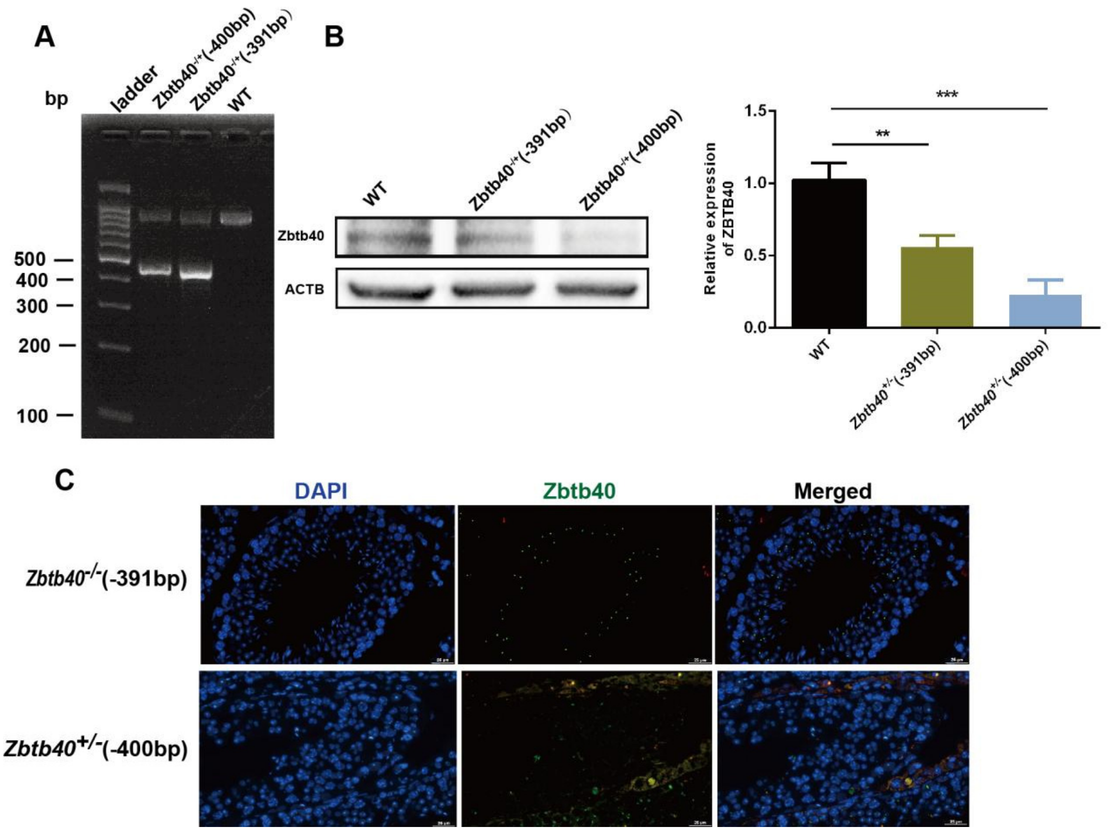

Figure S2. ZBTB40 expression in the *Zbtb40*$^{+/-}$ mice. (**A-C**) The KO efficiency of Zbtb40 at mRNA and protein levels in mouse testes were assessed by PCR (A), Western blots (B), and immunohistochemistry (C). *Zbtb40*$^{+/-}$#4 was the representative mice whose sequence of *Zbtb40* 400bp was lost, while *Zbtb40*$^{+/-}$#98 was the representative

mice whose sequence of *Zbtb40* 391bp was lost. ** indicated p<0.01 vs. the WT mice; *** denoted p<0.001 vs. the WT mice.

Figure S3

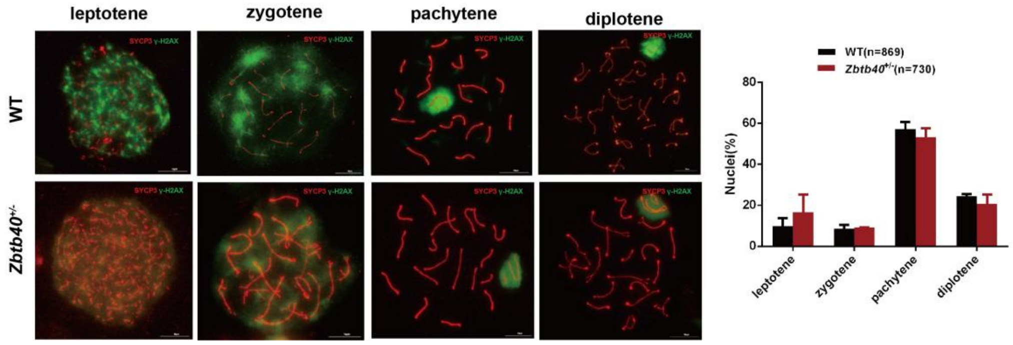

Figure S3. The influence of *Zbtb40* knockout on the number of the leptotene, zygotene, pachytene, and diplotene spermatocytes. SYCP3 (red fluorescence) and γH2AX (green fluorescence) were used to characterize the leptotene, zygotene, pachytene, and diplotene spermatocytes in the *Zbtb40*$^{+/-}$ mice and the WT mice.

Supplemental Tables 1-4

Table S1. Primers for PCR of ZBTB40 knockout mouse validation

Primers	Sequences (5'-3')	Tm (℃)	Product (bp)
Zbtb40-S	GGTTGCTGTTATTGGATGAG	53-61	850
Zbtb40-A	GCTTCTGTATTGACTTCTTTCG		

Table S2. Primary antibodies used in this study

Antibodies	Cat. No.#	Companies	Assays	Host
ZBTB40	abs134922-1	Absin	WB, IHC, IF/ICC, ELISA	Rabbit
ZBTB40	A301-932A	BETHYL	WB, ChIP, IHC, IF,	Rabbit
γ-H2AX	05-636	millipore	ICC, IF, WB, ChIP, IHC	Mouse
SYCP3	Ab15093	abcam	IHC-P, ICC/IF	Rabbit
FITC-PSA	L0770	Sigma	Acrosome status, sperm motility	
α-Tubulin	11H10	Cell signaling technology	WB, IHC, IF, F, IP, ChIP	Rabbit
Ki67	ab15580	abcam	IHC-P, ICC	Rabbit
VASA	ab27591	Abcam	ICC/IF, IHC-P, IHC-Fr, WB	mouse
β-actin	AP0060	Bioworld	WB	Rabbit
GAPDH	TA-08	Zhongshanjinqiao	WB	Rabbit

Table S3. Secondary antibodies utilized in this study

Antibodies	Companies	cat#	Reactivity
Goat Anti-Mouse HRP	Beyotime Biotechnology	A0126	Mouse
Goat Anti-Rabbit HRP	Beyotime Biotechnology	A0208	Rabbit
Alexa Fluor 488	Thermo scientific	A21202	Mouse
Alexa Fluor 555	Thermo scientific	A31572	Rabbit
Alexa Fluor 555	Thermo scientific	A31570	Mouse
Alexa Fluor 488	Thermo scientific	A21206	Rabbit

Table S4. Sequences of telomeric oligonucleotides

Telomere DNA	Oligonucleotide sequences 5'-3'
TTAGGG_ forward	TTAGGGTTAGGGTTAGGGTTAGGGTTAGGGTTAGGGTTAGGGTTAGGGTTAGGGTTAGGG
TTAGGG_ reverse	AACCCTAACCCTAACCCTAACCCTAACCCTAACCCTAACCCTAACCCTAACCCTAACCCT
